# Anti-Inflammatory and Antiosteoarthritis Effects of *Saposhnikovia divaricata ethanol* Extract: *In Vitro* and *In Vivo* Studies

**DOI:** 10.1155/2016/1984238

**Published:** 2016-03-02

**Authors:** Jin Mi Chun, Hyo Seon Kim, A Yeong Lee, Seung-Hyung Kim, Ho Kyoung Kim

**Affiliations:** ^1^K-herb Research Center, Korea Institute of Oriental Medicine, 1672 Yuseong-daero, Yuseong-gu, Daejeon 34054, Republic of Korea; ^2^Institute of Traditional Medicine and Bioscience, Daejeon University, Daejeon 34520, Republic of Korea; ^3^Mibyeong Research Center, Korea Institute of Oriental Medicine, 1672 Yuseong-daero, Yuseong-gu, Daejeon 34054, Republic of Korea

## Abstract

*Saposhnikovia divaricata* Schischkin has been used in traditional medicine to treat pain, inflammation, and arthritis. The aim of this study was to investigate the anti-inflammatory and antiosteoarthritis activities of* Saposhnikovia divaricata* extract (SDE). The anti-inflammatory effect of SDE was evaluated* in vitro* in lipopolysaccharide- (LPS-) treated RAW 264.7 cells. The antiosteoarthritic effect of SDE was investigated in an* in vivo* rat model of monosodium iodoacetate- (MIA-) induced osteoarthritis (OA) in which rats were treated orally with SDE (200 mg/kg) for 28 days. The effects of SDE were assessed* in vivo* by histopathological analysis and by measuring weight-bearing distribution, cytokine serum levels, and joint tissue inflammation-related gene expression. SDE showed anti-inflammatory activity by inhibiting the production of nitric oxide (NO), prostaglandin E_2_ (PGE_2_), tumor necrosis factor-*α* (TNF-*α*), and interleukin-6 (IL-6) in LPS-induced RAW 264.7 cells. In addition, SDE promoted recovery of hind limb weight-bearing, inhibited the production of proinflammatory cytokines and mediators, and protected cartilage and subchondral bone tissue in the OA rat model. Therefore, SDE is a potential therapeutic agent for OA and/or associated symptoms.

## 1. Introduction

Osteoarthritis (OA) is the most frequent musculoskeletal disorder and the most common degenerative joint disease in the elderly [1]. OA is a condition caused in part by injury, loss of cartilage structure and function, and dysregulation of proinflammatory and anti-inflammatory pathways [2, 3]. It primarily affects the articular cartilage and subchondral bone of synovial joints and results in joint failure, leading to pain upon weight-bearing including walking and standing [4].

There is no cure for OA, as it is very difficult to restore the cartilage once it is destroyed [5]. The goals of treatment are to relieve pain, maintain or improve joint mobility, increase the strength of the joints, and minimize the disabling effects of the disease. Pharmacological treatments of OA aim to reduce pain in order to increase the patient's joint function and quality of  life. Although cartilage destruction is the main event in OA, the degradation of collagen is the fundamental incident that determines the irreversible progression of OA in association with inflammation [6, 7]. Treatments with anti-inflammatory and chondroprotective activity are expected to relieve pain and maintain matrix integrity in OA patients.

Therefore, decreasing inflammation will likely be beneficial in OA management. Recent studies suggest protective roles for herbal resources on the progression of OA, in terms of mitigating chondrocyte inflammation and further cartilage destruction, through their ability to interact with joint-associated tissues, resulting in the mitigation of joint pain [8].

The root of* Saposhnikovia divaricata* Schischkin (Umbelliferae) has been widely used in traditional medicine for the treatment of headache, pain, inflammation, and arthritis in Korea and China [9, 10]. The diverse pharmacological effects of* Saposhnikovia divaricata* (SD) also include anti-inflammatory, analgesic, antipyretic, and antiarthritic properties [9, 11]. A recent study demonstrated that SD chromone extract possesses potential antirheumatoid arthritis effects in a mouse model of collagen-induced arthritis [10]; however, few studies have been conducted to support the anti-inflammatory and antiarthritis activity of* Saposhnikovia divaricata* extract (SDE).

Therefore, the present study investigated the anti-inflammatory and antiosteoarthritis activities of a 70% ethanol extract of SD. First, the anti-inflammatory effect of SDE was evaluated* in vitro* in LPS-induced RAW 264.7 cells. Next, the antiosteoarthritis effect of SDE was measured by assessing weight-bearing distribution, degradation of articular cartilage, and inflammatory responses in a rat model of monosodium iodoacetate- (MIA-) induced OA.

## 2. Materials and Methods

### 2.1. Preparation of SDE

The rhizomes of SD were purchased as a dried herb from Hanherb Co. (Guri, Korea). The plant materials were confirmed taxonomically by Dr. Go-Ya Choi of the Korea Institute of Oriental Medicine (KIOM). A voucher specimen (number 2014 SDE-6) was deposited in the Korean Herbarium of Standard Herbal Resources. Dried rhizomes of SD (320 g) were extracted twice with 70% ethanol (with a 2 h reflux) and the extract was then concentrated under reduced pressure. The decoction was filtered, lyophilized, and stored at 4°C. The yield of dried extract from crude starting materials was 48.13% (w/w).

### 2.2. Quantitative High-Performance Liquid Chromatography (HPLC) Analysis

Chromatographic analysis was performed with a HPLC system (Waters Co., Milford, MA, USA) and a photodiode array detector. For the HPLC analysis of SDE, the prim-*O*-glucosylcimifugin standard was purchased from the Korea Promotion Institute for Traditional Medicine Industry (Gyeongsan, Korea), and* sec-O*-glucosylhamaudol and 4′-*O*-*β*-D-glucosyl-5-*O*-methylvisamminol were isolated within our laboratory and identified by spectral analyses, primarily by NMR and MS.

SDE samples (0.1 mg) were dissolved in 70% ethanol (10 mL). Chromatographic separation was performed with an XSelect HSS T3 C18 column (4.6 × 250 mm, 5 *μ*m, Waters Co., Milford, MA, USA). The mobile phase consisted of acetonitrile (A) and 0.1% acetic acid in water (B) at a flow-rate of 1.0 mL/min. A multistep gradient program was used as follows: 5% A (0 min), 5–20% A (0–10 min), 20% A (10–23 min), and 20–65% A (23–40 min). The detection wavelength was scanned at 210–400 nm and recorded at 254 nm. The injection volume was 10.0 *μ*L. Standard solutions for the determination of three chromones were prepared at a final concentration of 7.781 mg/mL (prim-*O*-glucosylcimifugin), 31.125 mg/mL (4′-*O*-*β*-D-glucosyl-5-*O*-methylvisamminol), and 31.125 mg/mL (*sec-O*-glucosylhamaudol) in methanol and kept at 4°C.

### 2.3. Evaluation of Anti-Inflammatory Activity* In Vitro*

#### 2.3.1. Cell Culture and Sample Treatment

RAW 264.7 cells were obtained from the American Type Culture Collection (ATCC, Manassas, VA, USA) and grown in DMEM medium containing 1% antibiotics and 5.5% FBS. Cells were incubated in a humidified atmosphere of 5% CO_2_ at 37°C. To stimulate the cells, the medium was replaced with fresh DMEM medium, and lipopolysaccharide (LPS, Sigma-Aldrich Chemical Co., St. Louis, MO, USA) at 1 *μ*g/mL was added in the presence or absence of SDE (200 or 400 *μ*g/mL) for an additional 24 h.

#### 2.3.2. Determination of Nitric Oxide (NO), Prostaglandin E_2_ (PGE_2_), Tumor Necrosis Factor-*α* (TNF-*α*), and Interleukin-6 (IL-6) Production

Cells were treated with SDE and stimulated with LPS for 24 h. NO production was analyzed by measuring nitrite using the Griess reagent according to a previous study [12]. Secretion of the inflammatory cytokines PGE_2_, TNF-*α*, and IL-6 was determined using an ELISA kit (R&D systems) according to manufacturer instructions. The effects of SDE on NO and cytokine production were determined at 540 nm or 450 nm using a Wallac EnVision*™* microplate reader (PerkinElmer).

### 2.4. Evaluation of Antiosteoarthritis Activity* In Vivo*

#### 2.4.1. Animals

Male Sprague-Dawley rats (7 weeks old) were purchased from Samtako Inc. (Osan, Korea) and housed under controlled conditions with a 12-h light/dark cycle at 22 ± 2°C and 55 ± 15% humidity. Rats were provided with a laboratory diet and water* ad libitum*. All experimental procedures were performed in compliance with the National Institutes of Health (NIH) guidelines and approved by the Animal Care and Use Committee of the Daejeon university (Daejeon, republic of Korea).

#### 2.4.2. Induction of OA with MIA in Rats

The animals were randomized and assigned to treatment groups before the initiation of the study (*n* = 6 per group). MIA solution (3 mg/50 *μ*L of 0.9% saline) was directly injected into the intra-articular space of the right knee under anesthesia induced with a mixture of ketamine and xylazine. Rats were divided randomly into four groups: (1) the saline group with no MIA injection, (2) the MIA group with MIA injection, (3) the SDE-treated group (200 mg/kg) with MIA injection, and (4) the indomethacin- (IM-) treated group (2 mg/kg) with MIA injection. Rats were administered orally with SDE and IM 1 week before MIA injection for 4 weeks. The dosage of SDE and IM used in this study was based on those employed in previous studies [10, 13, 14].

#### 2.4.3. Measurements of Hindpaw Weight-Bearing Distribution

After OA induction, the original balance in weight-bearing capability of hindpaws was disrupted. An incapacitance tester (Linton instrumentation, Norfolk, UK) was used to evaluate changes in the weight-bearing tolerance. Rats were carefully placed into the measuring chamber. The weight-bearing force exerted by the hind limb was averaged over a 3 s period. The weight distribution ratio was calculated by the following equation: [weight on right hind limb/(weight on right hind limb + weight on left hind limb)] × 100 [15].

#### 2.4.4. Measurements of Serum Cytokine Levels

The blood samples were centrifuged at 1,500 g for 10 min at 4°C; then the serum was collected and stored at −70°C until use. The levels of IL-1*β*, IL-6, TNF-*α*, and PGE_2_ in the serum were measured using ELISA kits from R&D Systems (Minneapolis, MN, USA) according to manufacturer instructions.

#### 2.4.5. Real-Time Quantitative RT-PCR Analysis

Total RNA was extracted from knee joint tissue using the TRI reagent® (Sigma-Aldrich, St. Louis, MO, USA), reverse-transcribed into cDNA and PCR-amplified using a TM One Step RT PCR kit with SYBR green (Applied Biosystems, Grand Island, NY, USA). Real-time quantitative PCR was performed using the Applied Biosystems 7500 Real-Time PCR system (Applied Biosystems, Grand Island, NY, USA). The primer sequences and the probe-sequence are shown in Table 1. Aliquots of sample cDNAs and an equal amount of GAPDH cDNA were amplified with the TaqMan® Universal PCR master mixture containing DNA polymerase according to manufacturer instructions (Applied Biosystems, Foster, CA, USA). PCR conditions were 2 min at 50°C, 10 min at 94°C, 15 s at 95°C, and 1 min at 60°C for 40 cycles. The concentration of target gene was determined using the comparative Ct (threshold cycle number at cross-point between amplification plot and threshold) method, according to manufacturer instructions.

#### 2.4.6. Histopathological Analysis

Tissue specimens from the knee joint of rats were removed, fixed in 10% formalin, embedded in paraffin, and serially sectioned at 7 *μ*m. Tissue sections were then stained with hematoxylin and eosin (H&E) or Safranin O-fast green. Histological changes were examined by light microscopy (Olympus CX31/BX51, Olympus Optical Co., Tokyo, Japan) and photographed (Olympus DP70).

### 2.5. Statistical Analysis

All results are presented as the mean ± standard deviation (SD). The statistical analysis was performed using one-way analysis of variance (ANOVA), Duncan's multiple range test was performed to identify significant differences between groups, and *p* values of < 0.05 were considered to be statistically significant.

## 3. Results

### 3.1. Chemical Profile of SDE

**Figure 1:**
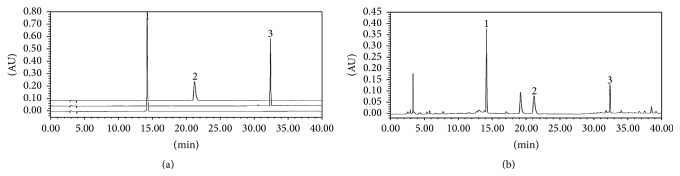
HPLC chromatograms of standard compounds (a) and SDE (b). (1) Prim-*O*-glucosylcimifugin, (2) 4′-*O*-*β*-D-glucosyl-5-*O*-methylvisamminol, and (3)* sec-O*-glucosylhamaudol.

To identify and quantify the levels of marker components in SDE, HPLC analysis was performed. The chromatogram of the main components is shown in Figure 1. The three components of SDE, prim-*O*-glucosylcimifugin, 4′-*O*-*β*-D-glucosyl-5-*O*-methylvisamminol, and* sec-O*-glucosylhamaudol, were detected at approximately 14.2, 21.2, and 32.3 min, respectively. The prim-*O*-glucosylcimifugin content was the highest (1.07%), followed by 4′-*O*-*β*-D-glucosyl-5-*O*-methylvisamminol (0.75%) and* sec-O*-glucosylhamaudol (0.21%).

### 3.2. Effect of SDE on NO, PGE_2_, TNF-*α*, and IL-6 Production* In Vitro*

**Figure 2:**
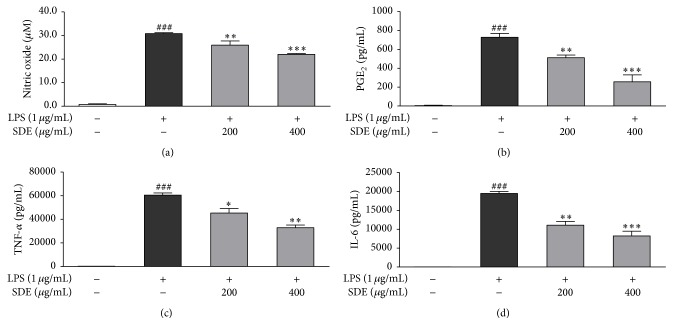
Effects of SDE on the production of cytokines and inflammatory mediators in LPS-stimulated RAW 264.7 macrophages. Cells were treated with SDE (0, 200, or 400 *μ*g/mL) plus LPS (1 *μ*g/mL) or LPS alone for 24 h. (a) NO production was measured using the Griess reagent. (b–d) Production of PGE_2_, TNF-*α*, and IL-6 was measured by ELISA. Values are expressed as the means ± SD (*n* = 3). ^###^ *p* < 0.001 versus untreated LPS and SDE; ^*∗*^ *p* < 0.05, ^*∗∗*^ *p* < 0.01, and ^*∗∗∗*^ *p* < 0.001 versus LPS alone.

We examined the effects of SDE on the levels of NO, PGE_2_, TNF-*α*, and IL-6 in LPS-stimulated RAW 264.7 cells. Cells were treated with SDE plus LPS or LPS alone for 24 h. SDE significantly inhibited the production of NO, PGE_2_, TNF-*α*, and IL-6 at a range of 200 or 400 *μ*g/mL (Figure 2). In addition, SDE did not affect cell viability and was not toxic to RAW 264.7 cells (data not shown).

### 3.3. Effect of SDE on Changes in Hindpaw Weight-Bearing Distribution

**Figure 3:**
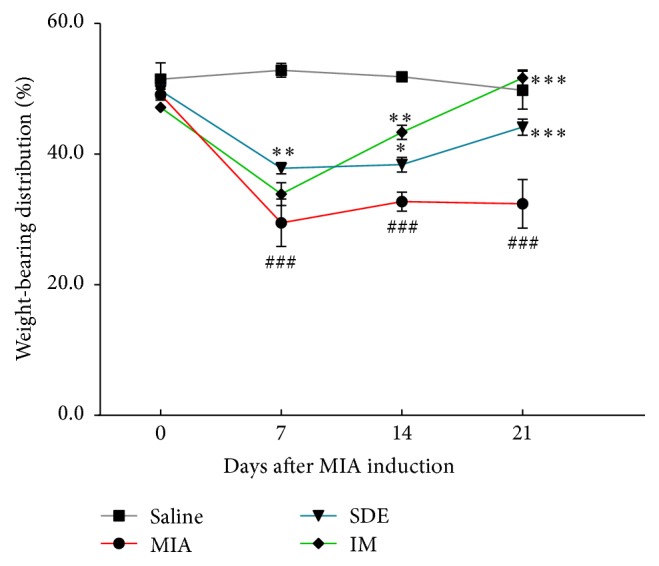
Effects of SDE on changes in hindpaw weight-bearing distribution in MIA-induced OA in rats. The weight-bearing distribution ratio was measured once a week for 21 days after the injection of MIA using an incapacitance tester, compared to that of the MIA-induced group. ^###^ *p* < 0.001 versus saline; ^*∗*^ *p* < 0.05, ^*∗∗*^ *p* < 0.01, and ^*∗∗∗*^ *p* < 0.001 versus MIA.

Weight distribution was measured between sensitized and contralateral hind limbs and used as an index of joint discomfort in the arthritic knee. Therefore, we evaluated hindpaw weight-bearing using an incapacitance tester for 21 days. The ratio of hindpaw weight distribution between the right and left limbs was used to assess the progression of OA [16]. The weight-bearing distribution of the MIA group reduced rapidly and became significantly different from that of the saline group by day 7 post-MIA injection and was maintained for at least 21 days. By contrast, in the SDE- and IM-treated groups, these values were only slightly decreased at day 7 compared with those of the MIA group. Beyond that, there was full recovery and the balance between both hind legs returned to normal in the SDE- and IM-treated groups. These results demonstrate significant recovery of hind limb weight-bearing in the SDE-treated group (Figure 3).

### 3.4. Effect of SDE on Inflammatory Cytokine Serum Levels

**Figure 4:**
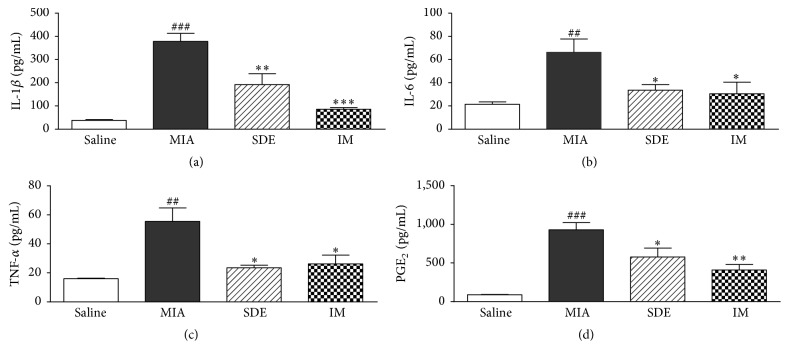
Effects of SDE on serum levels of cytokines and inflammatory mediators in MIA-induced OA in rats. (a) Serum IL-1*β*, (b) IL-6, (c) TNF-*α*, and (d) PGE_2_ levels were measured by ELISA. ^##^ *p* < 0.01 and ^###^ *p* < 0.001 versus saline; ^*∗*^ *p* < 0.05, ^*∗∗*^ *p* < 0.01, and ^*∗∗∗*^ *p* < 0.001 versus MIA.

Proinflammatory cytokines have important roles in the maintenance of chronic inflammation and tissue damage during the progression of OA. Therefore, we investigated the effects of SDE on the serum levels of IL-1*β*, IL-6, TNF-*α*, and PGE_2_ in the MIA-induced OA model. The serum levels of IL-1*β*, IL-6, TNF-*α*, and PGE_2_ increased in the MIA group compared with those in the saline group but were suppressed in the SDE- and IM-treated groups compared with those in the MIA group (Figure 4). These results indicate that SDE might have cartilage protection effects in the MIA model by regulating inflammatory cytokines.

### 3.5. Expression of Cytokine and Inflammatory Mediator mRNA in Knee Joint Tissues

**Figure 5:**
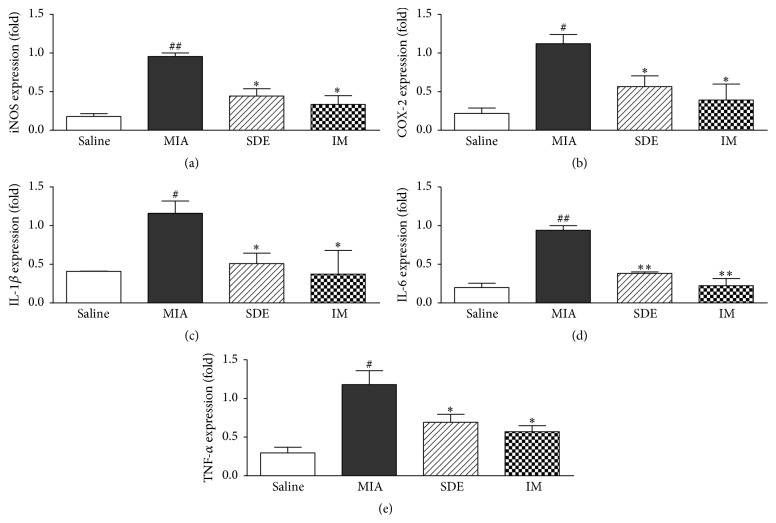
Effects of SDE on the expression of cytokines and inflammatory mediators in the knee joint of rats with MIA-induced OA. Expression of (a) iNOS, (b) COX-2, (c) IL-1*β*, (d) IL-6, and (e) TNF-*α* mRNA was determined by real-time RT-PCR. ^#^ *p* < 0.05 and ^##^ *p* < 0.01 versus saline; ^*∗*^ *p* < 0.05 and ^*∗∗*^ *p* < 0.01, versus MIA.

We investigated the effects of SDE on the mRNA levels of COX-2, iNOS, IL-1*β*, IL-6, and TNF-*α* in the knee joint tissues of rats. We also investigated the effects of SDE on the mRNA levels of cytokines in the knee joint tissues of rats. The expression of cytokine and inflammatory mediator mRNAs increased following MIA injection, but the expression of the cytokine was attenuated in the SDE- and IM-treated groups (Figure 5). Thus, our results indicate that SDE suppresses the expression of inflammatory cytokines in MIA-treated rats.

### 3.6. Effects of SDE on Histopathology

**Figure 6:**
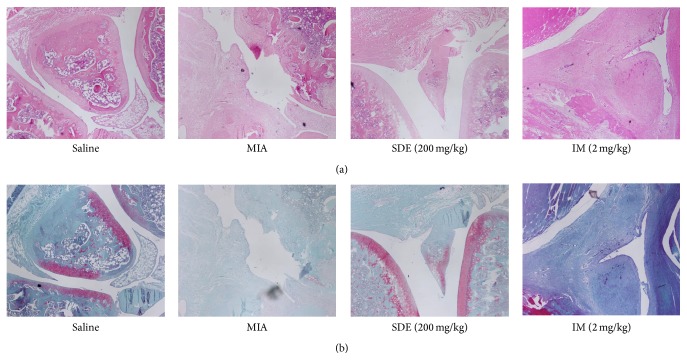
Histopathological features of the knee joint tissues of rats with MIA-induced OA. Representative photographs of knee joint tissues stained with (a) H&E or (b) Safranin O-fast green (magnification, 100x).

Following sacrifice of the rats, the knee joints were evaluated histologically for severity of inflammation, synovial hyperplasia, and bone damage by H&E and Safranin O staining. The MIA group exhibited histological changes indicative of severe arthritis, with extensive infiltration of inflammatory cells into articular tissues, exudation into the synovial space, synovial hyperplasia, and cartilage erosion; however, treatment with SDE and IM inhibited the damage and synovial hyperplasia in joints (Figure 6). These histological features show that SDE attenuates the severity of MIA-induced OA in rats.

## 4. Discussion

The current available treatments for OA focus on target symptom reduction, maintenance of joint mobility, and limitation of the loss of functional capacity. Many studies suggest that traditional herbal resources benefit the management of inflammatory arthritis and may therefore benefit OA [17–19]. SD shows several medicinal therapeutic effects; however, to date, no studies have been conducted to evaluate the efficacy of SD for the treatment of OA. Therefore, the present study was conducted to evaluate the anti-inflammatory and antiosteoarthritic activities of SDE using LPS-induced macrophages and a MIA-induced OA model.

Inflammatory mediators play key roles in the progression of cartilage destruction in OA [18]. The proinflammatory cytokines are deemed to display catabolic properties that influence the pathophysiological processes of OA [20]. Our results show that SDE inhibits the production of NO, PGE_2_, TNF-*α*, and IL-6 in LPS-stimulated RAW 264.7 cells.

The antiosteoarthritic effect of SDE in MIA-induced rats was evaluated by measuring weight-bearing distribution, inflammatory cytokines, and mediators in serum, inflammation-related gene expression in knee joints, and histopathological parameters. In this study, we also provide evidence for an OA-related pain-relieving effect of SDE in the MIA OA model. OA pain can be triggered by joint movement and typically results in diminished use and reduced joint mobility [21]. Our results show that SDE significantly protects weight-bearing in MIA-induced OA in rats, suggesting that SDE could be useful for treating OA pain.

The chondroprotective effects of SDE, via reduction of inflammation, have been established in rheumatoid arthritis animal models [10]. Consistent with published work, the present study demonstrated that SDE exerts chondroprotective effects in MIA-induced OA in rats by suppressing the expression of inflammatory cytokines and mediators in serum and inflammation-related genes in knee joints.

OA is a condition caused in part by injury loss of cartilage structure and function and dysregulation of proinflammatory and anti-inflammatory pathways [3, 12]. Inflammation is an important factor associated with the development and progression of OA. Catabolic and proinflammatory mediators, for example, cytokines, NO, and PGE_2_, are produced by inflamed synovium and alter the balance of cartilage matrix degradation and repair. These processes will then exacerbate clinical symptoms and joint degradation in OA [22]. Therefore, inhibiting proinflammatory cytokines could be an important approach to managing OA. In this study, SDE may inhibit inflammatory reactions and partially prevent and slow the progression of OA. In addition, we demonstrated that SDE attenuates histological damage and synovial hyperplasia in joints, compared with MIA group. These results suggest that SDE prevents the degradation of cartilage and cartilage inflammation, resulting in the prevention of OA progression.

SD contains major bioactive constituents, including chromones such as prim-*O*-glucosylcimifugin, 4′-*O*-*β*-D-glucosyl-5-*O*-methylvisamminol, cimifugin, and* sec-O*-glucosylhamaudol, which are usually obtained from the roots of the plant [10, 23, 24]. Prim-*O*-glucosylcimifugin, the chromone with the highest content in the roots of SD, showed significant anti-inflammatory effects on LPS-induced inflammatory responses in RAW 264.7 cells and significantly protected mice against LPS-induced acute lung injury [25]. SD ethanol extract and chromones isolated from SD showed potential anti-inflammatory and protective effects in LPS-activated RAW 264.7 cells [11, 26]. In the present study, we confirmed the presence of the chromones, which were prim-*O*-glucosylcimifugin, 4′-*O*-*β*-D-glucosyl-5-*O*-methylvisamminol, and* sec-O*-glucosylhamaudol in SDE, and this partially explains the anti-inflammatory activity of SDE.

## 5. Conclusions

In conclusion, SDE showed anti-inflammatory activity by inhibiting the production of NO, PGE_2_, TNF-*α*, and IL-6 in LPS-induced RAW 264.7 cells. SDE also attenuated joint pain and stiffness, inhibited the production of proinflammatory cytokines and mediators, and protected cartilage and subchondral bone tissue in an OA rat model. Therefore, our results suggest that SDE may be a potentially suitable therapeutic agent for OA and/or its associated symptoms.

## Figures and Tables

**Table 1:** Real-time PCR primer sequences.

Gene		Primer sequence
IL-1*β*	Forward	5′-CCCTGCAGCTGGAGAGTGTGG-3′
	Reverse	5′-TGTGCTCTGCTTGAGAGGTGCT-3′

IL-6	Forward	5′-TTCCTACCCCAACTTCCAATG-3′
	Reverse	5′-ATGAGTTGGATGGTCTTGGTC-3′

TNF-*α*	Forward	5′-GACCCTCACACTCAGATCATCTTCT-3′
	Reverse	5′-TGCTACGACGTGGGCTACG-3′

NOS-II	Forward	5′-CTTTACGCCACTAACAGTGGCA-3′
	Reverse	5′-AGTCATGCTTCCCATCGCTC-3′

COX-2	Forward	5′-TGGTGCCGGGTCTGATGATG-3′
	Reverse	5′-GCAATGCGGTTCTGATACTG-3′

GAPDH	Probe	Applied Biosystems® Rat GAPD (GAPDH) Endogenous Control (VIC®/MGB Probe, 4352338E)
